# Supplementary material for: TBCC Domain-Containing Protein Regulates Sporulation and Virulence of Phytophthora capsici via Nutrient-Responsive Signaling
Source: Int J Mol Sci. 2024 Nov 16;25(22):12301. doi: 10.3390/ijms252212301 (PMC11595192; doi:10.3390/ijms252212301)
Supplement: Supplementary file 1 [file ijms-25-12301-s001.zip › ijms-3258776-supplementary.pdf]

TBCC domain-containing protein regulates sporulation and virulence of *Phytophthora capsici* via nutrient-responsive signaling

Authors: Yaru Guo[1*], Xiang Qiu[1*], Caihuan Ou,[1] Bingting Lai[1], Hengyuan Guo[1], Huirong Wang[1], Linying Li[1], Lili Lin[2], Dan Yu[1], Wenbo Liu, Justice Norvienyeku[1#]

[1]Key Laboratory of Green Prevention and Control of Tropical Diseases and Pests, Ministry of Education, School of Tropical Agriculture and Forestry, Hainan University, Haikou 570228, China.
[2]Ministerial and Provincial Joint Innovation Centre for Safety Production of Cross-Strait Crops, Fujian Agriculture and Forestry University, Fuzhou 350002, China

*These authors contributed equally to this work
[#]**Corresponding author:** Norvienyeku Justice
School of Tropical Agriculture and Forestry, Hainan University, Haikou 570228, China.
E-mail: jk_norvienyeku@hainanu.edu.cn
ORCID: 0000-0002-0889-7233

Supplementary Figures and Tables

Table S1. List of primers used in this study

Primer Name	Sequence
Tbcc-qPCR-F	5' TACCTCTATTCGCTCACG 3'
Tbcc-qPCR-R	5' GGGTCATTGAAGTCGTAG 3'
Tbcc-AF	5' GCTGCTCTTCTCTACTAC 3'
Tbcc-AR	5' CTCTCCCTGACCATTATTG 3'
Tbcc-BF	5' CGGAATAGGAATGCTGAGA 3'
Tbcc-BR	5' GACGGTGCTTGTTCAATC 3'
Tbcc-OF	5' CGCATGAGTTGATAGAC 3'
Tbcc-OR	5' CGAATGTACGATCAGTC 3'
Tbcc-Seq-F:	5' GTCCTTTGATAGCGATGG 3'
Tbcc-Seq-R:	5' CTCAGCATTCCTATTCCG 3'
Tbcc- Qu-F:	5' GTCCTTTGATAGCGATGG 3'
Tbcc- Qu-R:	5' TAGCAGAAGAGTGGGAAG 3'
Tbcc-Comp-F	5' AAGCTTCTACTACGTCATC 3'
Tbcc-Comp-R	5' CATCATGCCTATCATTTCTG 3'
Tbcc-GFP-F	5' ATGGGCAAGGGCGAGGAA 3'
Tbcc-GFP-R	5' TCACTTGTAGAGTTCATCCATGCC 3'
pvx-R:	5' GTGGCTGTTGTAGTTGT 3'
Tbcc-Pvx-F:	5' ATGATTTTCACTGCGTCGGATGG 3'
Tbcc-Pvx-R:	5' CATCATGCCTATCATTTCTGTCA 3'
Tbcc ALL-F:	5' CAATAACCTCGGGAACAG 3'
Tbcc ALL-R:	5' CTGGAAGACATGACACTC 3'

Tbcc-sg1-F	5'CTAGCGCGTCTCTGATGAGTCCGTGAGGACGAAACGA GTAAGCTCGTCAGACGCTCATGAAGCTTCCG 3'
Tbcc-sg1-R	5' AAACCGGAAGCTTCATGAGCGTCTGACGAGCTTAC TCGTTTCGTCCTCACGGACTCATCAGAGACGCG 3'
Tbcc-sg2-F	5' CTAGCAAATGGCTGATGAGTCCGTGAGGACGAAAC GAGTAAGCTCGTCCCATTTAACGGAGCTTACTG 3'
Tbcc-sg2-R	5 'AAACCAGTAAGCTCCGTTAAATGGGACGAGCTTA CTCGTTTCGTCCTCACGGACTCATCAGCCATTTG 3'
Tbcc-sg3-F	5' CTAGCGATGGTCTGATGAGTCCGTGAGGACGAAA CGAGTAAGCTCGTCACCATCTACAGTGCCTCCAGT 3'
Tbcc-sg3-R	5' AAACACTGGAGGCACTGTAGATGGTGACGAGCTTAT CGTTTCGTCCTCACGGACTCATCAGACCATCG 3'
G418-F	5' TCAGAAGAACTCGTCAAGAA 3'
G418-R	5' TCAGAAGAACTCGTCAAGAA 3'
HPH-F	5' TGTCAAGTCTCCACGAAG 3'
HPH-R	5' CTATTCCTTTGCCCTCGGA 3'
M13-F	5 'GTTGTAAAACGACGGCCAG 3'
M13-R	5 'CAGGAAACAGCTATGACC 3'

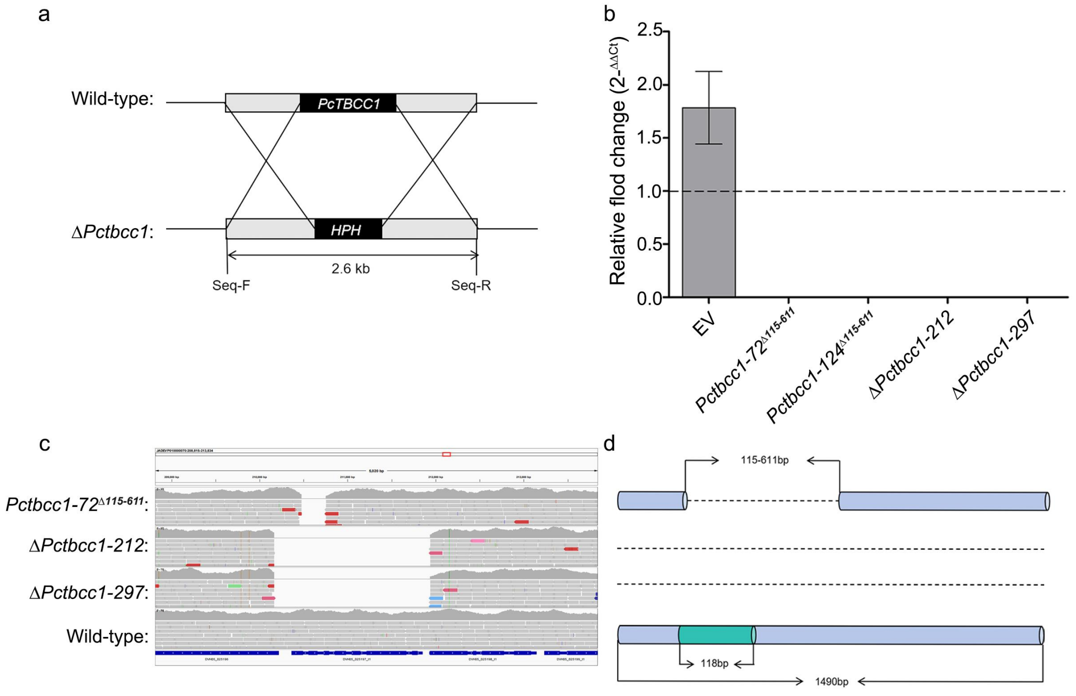

Figure S1: Generation and identification of PcTBCC-1 gene deletion strains (a) **S**howed the schematic representation of the PcTBCC obtained by the homologous recombination technique. (b) **S**howed the Expression level of TBCC was monitored in wild-type, Δ*Pctbcc1-212,* Δ*Pctbcc1-297, Pctbcc1-72*$^{\Delta 115\text{-}611}$, Δ*Pctbcc1-124*$^{\Delta 115\text{-}611}$. (c) Showed the Alignment plots of whole-genome sequencing of wild-type, Δ*Pctbcc1-212,* Δ*Pctbcc1-297, Pctbcc1-72*$^{\Delta 115\text{-}611}$, Δ*Pctbcc1-124*$^{\Delta 115\text{-}611}$. (d) Showed the Schematic structure of the deletion of *PcTBCC* gene in the Δ*Pctbcc1* strains compared to the wild-type strains.
